# BA inhibits LPS-stimulated inflammatory response and apoptosis in human middle ear epithelial cells by regulating the Nf-Kb/Iκbα axis

**DOI:** 10.1515/biol-2022-1019

**Published:** 2024-12-31

**Authors:** Qian He, Yanzhi Cai, Meihua Kong

**Affiliations:** Department of ENT, Yueqing People’s Hospital, 338, Qingyuan Road, Chengnan Street, Yueqing City, Wenzhou, Zhejiang, 325600, China

**Keywords:** boswellic acid, lipopolysaccharide, human middle ear epithelial cells, inflammation, NF-κB/IκBα axis

## Abstract

Otitis media (OM) is a prevalent childhood ear disease characterized by inflammation of the middle ear cavity, which can lead to ear pain, fever, and hearing loss. The pathogenesis of OM is multifaceted, encompassing a variety of factors including bacterial or viral infections, host immune responses, and the function of middle ear epithelial cells. Boswellic acid (BA), a natural triterpene compound extracted from frankincense resin, has been proven to possess significant anti-inflammatory and immunomodulatory effects. This study aims to investigate the effects of BA on lipopolysaccharide (LPS)-stimulated inflammatory responses and apoptosis in human middle ear epithelial cells (HMEECs) and its potential mechanisms. Our findings demonstrated that BA enhances the proliferation of LPS-stimulated HMEECs and concurrently inhibits their apoptosis. In addition, BA blocked LPS-stimulated HMEEC inflammation. Mechanistically, BA suppressed the NF-κB/IκBα axis in LPS-stimulated HMEECs. In conclusion, BA effectively inhibits LPS-stimulated inflammation and apoptosis by mediating the NF-κB/IκBα axis, highlighting its potential as a therapeutic agent for OM.

## 1 Introduction

Otitis media (OM) is a prevalent inflammatory disease that affects the middle ear cavity of children, posing a considerable threat to children’s health. This can lead to symptoms such as ear pain and may contribute to behavioral problems [1,2]. The absence of universally accepted diagnostic criteria makes it difficult to accurately determine the prevalence data of OM [3,4]. Middle ear inflammation, which can be triggered by various primary factors, plays a pivotal role in the pathogenesis of OM [5,6]. Persistent acute inflammatory responses or defects in immune regulation during intermediate stages of inflammation can exacerbate inflammatory processes [7]. However, overly robust inflammatory responses frequently culminate in immunopathology and tissue damage within the middle ear, consequently leading to conductive hearing loss [8]. Consequently, it is imperative to meticulously regulate excessive innate inflammatory responses. This underscores the necessity of discovering a new pharmacological treatment for OM.

In recent years, the interest in herbal medicines as alternative therapeutic agents and health supplements has significantly increased. Herbal medicines, traditionally derived from plant materials, have had their active components subjected to thorough scientific investigation. Among these, the multifaceted effects of boswellic acid (BA), a compound consisting of triterpenic acids sourced from the resin of *Boswellia serrata*, have received considerable scholarly attention. Specifically, research has focused on its anti-inflammatory, immunomodulatory, and anti-tumor properties and its efficacy in treating inflammatory bowel disease [9]. BA positively influences lysosomal acid hydrolase activity, lipid peroxidation, and antioxidant status in mice suffering from gouty arthritis [10,11]. In rats, BA can prevent bisphenol alpha- and gamma radiation-stimulated hepatic steatosis and cardiac remodeling [12]. BA synergizes with low-level ionizing radiation to mediate bisphenol-stimulated pulmonary toxicity in rats by inhibiting the JNK/ERK/c-Fos axis [13]. BA exhibits anti-inflammatory properties and simultaneously enhances the anti-tumor efficacy of temozolomide alongside the irreversible ErbB family blocker, afatinib, in glioblastoma cells [14,15]. *In vitro* studies demonstrate that BA effectively inhibits the induction of various inflammatory mediators that are mediated by IL-1β and TLR4 within osteoarthritis synovial explant tissues [16]. However, the specific role and mechanism of BA in the context of OM remain unclear.

This study seeks to bridge the existing gap by exploring the effects of BA specifically on lipopolysaccharide (LPS)-stimulated human middle ear epithelial cells (HMEECs), which act as a model for OM. Our focus is on the modulation of the NF-κB/IκBα axis, providing a novel insight into the therapeutic potential of BA in the context of OM.

## 2 Materials and methods

### 2.1 Cell culture and treatment

HMEECs were obtained from ATCC (ATCC^®^ CRL-2836™). The immortalized HMEECs were cultured in DMEM complete medium (Gibco, USA) and incubated at 37°C in an atmosphere containing 5% CO_2_. The cells were treated with LPS (100 ng/mL; Sigma-Aldrich, USA) for 24 h to induce inflammation. BA (Sigma-Aldrich, USA) was dissolved in dimethyl sulfoxide and applied at concentrations of 2.5, 5, and 10 μM for 24 h. All experiments were conducted in triplicate.

### 2.2 Cell viability assays

The HMEECs were cultured in 96-well plates and incubated at 37°C. Following the specified treatment for a duration of 24 h, cells were subsequently exposed to CCK-8 reagent at 37°C for a period of 4 h. The relative cell viability was assessed with a spectrophotometer at 450 nm wavelength (Bio-Rad, USA).

### 2.3 Edu assay

The HMEECs were incubated with Edu agent (ab219801; Abcam) for 2 h after which the agent was removed. Following this, the cells were photographed by a fluorescence microscope (Zeiss, German).

### 2.4 Flow cytometry (FCM) assay

The HMEECs were washed with PBS and fixed using 70% ethanol at −20°C for 2 h. Subsequently, the cells were stained with PI at 4°C. Then, the cells were detected using a flow cytometer (BD, USA).

### 2.5 Enzyme-linked immunosorbent assay (ELISA)

Following the specified stimulations, the supernatants from the cells were subjected to ELISA assay to quantify the concentrations of TNF-α, IL-1β, and IL-6 (Abcam).

### 2.6 Immunoblot

Protein was separated by 10% SDS-PAGE, and further transferred onto the PVDF membranes. The proteins were blocked with 5% milk for 1 h. Primary antibodies including Bax (1:500, ab32503; Abcam), Bcl-2 (1:500, ab182858; Abcam), cleaved caspase-3 (1:1,000, ab32042; Abcam), TNF-α (1:1,000, ab183218; Abcam), IL-6 (1:1,000, ab233706), IL-1β (1:500, ab216995), p-ERK1/2 (1:1,000, ab201015), ERK1/2 (1:1,000, ab184699), p-JNK (1:500, ab215208), JNK (1:500, ab110724), p-p38 (1:1000, ab17886), p38 (1:1,000, ab170099), p-p65 (1:500, ab76302; Abcam), p65 (1:1,000, ab32536; Abcam), p-IκBα (1:500, ab133462; Abcam), IκBα (1:500, ab32518; Abcam), and GAPDH (1:3,000; ab8245; Abcam), and secondary antibodies were incubated for 1 h and photographed after chemiluminescence. Immunoblot analyses were conducted in triplicate, and the bands’ intensities were quantified using ImageJ software. The relative expression levels of the proteins across various BA doses were normalized against the GAPDH control.

### 2.7 Statistical analysis

**Figure 1:**
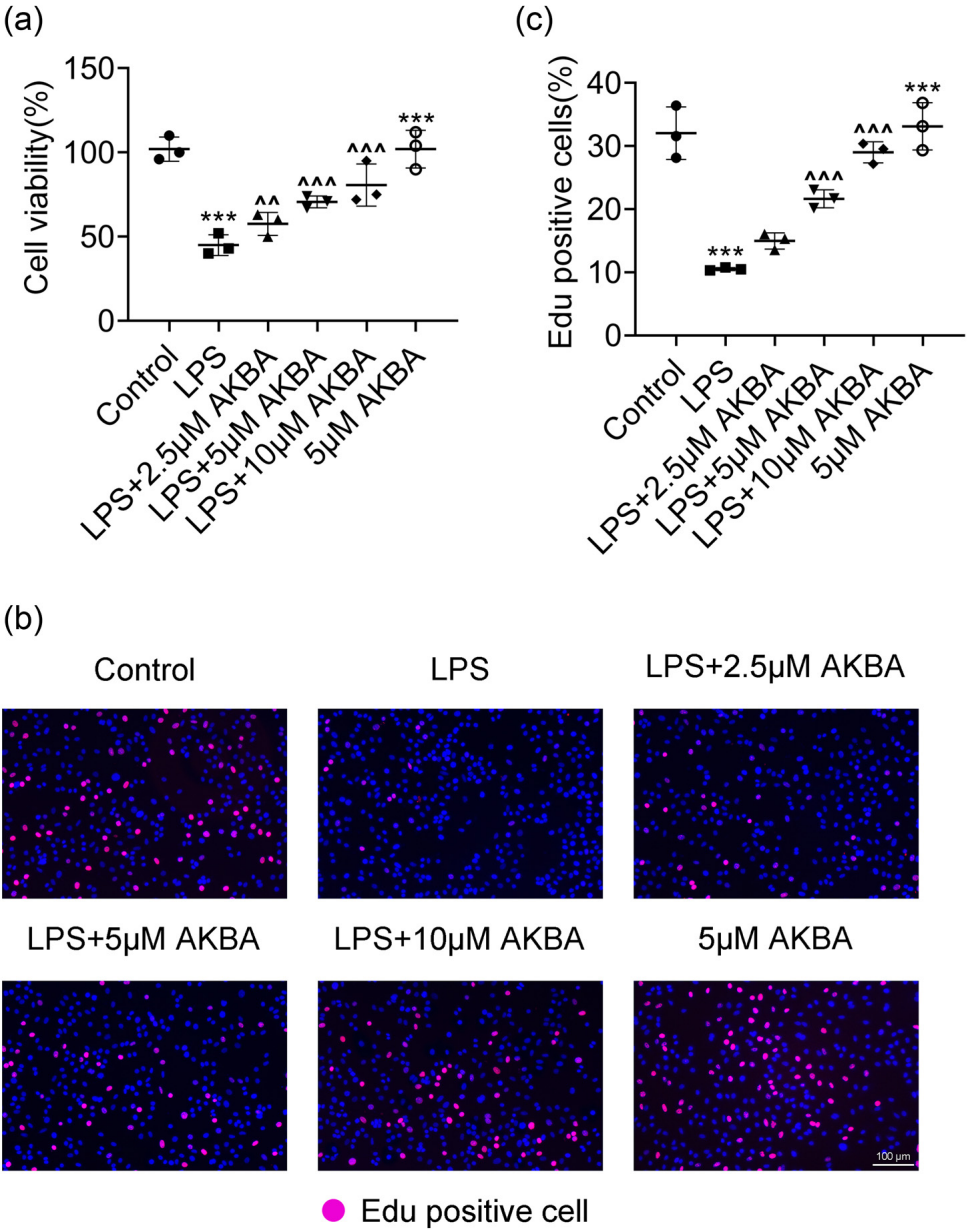
BA promotes the growth of LPS-stimulated HMEECs. (a) CCK-8 assays showed the growth of HMEECs upon LPS treatment and the treatment of BA at concentrations of 2.5, 5, and 10 μM or BA (5 μM) alone for 24 h. The OD450 value was measured (*n* = 3). (b) Edu assays showed the growth degree of HMEECs upon LPS treatment and the treatment of BA at concentrations of 2.5, 5, and 10 μM or BA (5 μM) alone for 24 h. Scale bar, 100 μm (*n* = 3). (c) Percentage of Edu-positive cells was quantified (*n* = 3). ***p < 0.001, LPS vs control, ^^*p* < 0.01, ^^^*p* < 0.001, LPS+AKBA vs LPS. AKBA, BA.

All experiments were performed in triplicate, and data are presented as mean ± standard deviation (SD). Group differences were analyzed using one-way ANOVA followed by *post-hoc* multiple comparison tests conducted using GraphPad 8.0 software. Statistical significance was set at *p* < 0.05. Error bars represent the standard deviation of each group, ensuring the accuracy and reproducibility of the results (Figure 1).

## 3 Results

### 3.1 BA promotes the growth of LPS-stimulated HMEECs

#### 3.1.1 BA inhibited the apoptosis of LPS-stimulated HMEECs

**Figure 2:**
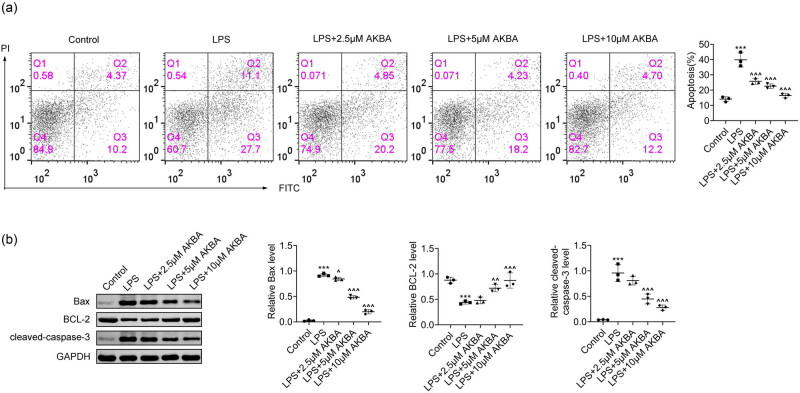
BA inhibited the apoptosis of LPS-stimulated HMEECs. (a) FCM assays showed the apoptosis degree of HMEECs upon LPS treatment and the treatment of BA at concentrations of 2.5, 5, and 10 μM for 24 h. The percentage of apoptosis cells was quantified (*n* = 3). (b) Immunoblot assays showed the expression of Bax, Bcl-2, and cleaved caspase-3 in HMEECs upon LPS treatment and the treatment of BA at concentrations of 2.5, 5, and 10 μM for 24 h. The relative expression levels were quantified (*n* = 3). ***p < 0.001, LPS vs control, ^*p* < 0.05, ^^*p* < 0.01, ^^^*p* < 0.001, LPS+AKBA vs LPS. AKBA, BA.

The impact of BA on the apoptosis of the OM cell model was evaluated. FCM assays demonstrated that LPS treatment, which simulates OM in HMEECs, significantly increased the apoptosis rates in HMEECs (Figure 2a). However, BA further inhibited apoptosis in LPS-stimulated HMEECs, evidenced by a reduced percentage of apoptotic cells (Figure 2a). In the immunoblot assays conducted, an elevation in the expression of Bax and cleaved caspase-3, alongside a reduction in Bcl-2 expression, was observed in HMEECs following LPS stimulation. Conversely, the administration of BA to LPS-stimulated HMEECs was associated with a decreased expression of Bax and cleaved caspase-3, while concurrently enhancing Bcl-2 expression. These findings suggest an inhibitory effect on apoptosis, as depicted in Figure 2b. Therefore, BA inhibited the apoptosis of LPS-stimulated HMEECs.

### 3.2 BA blocked LPS-stimulated HMEEC inflammation

**Figure 3:**
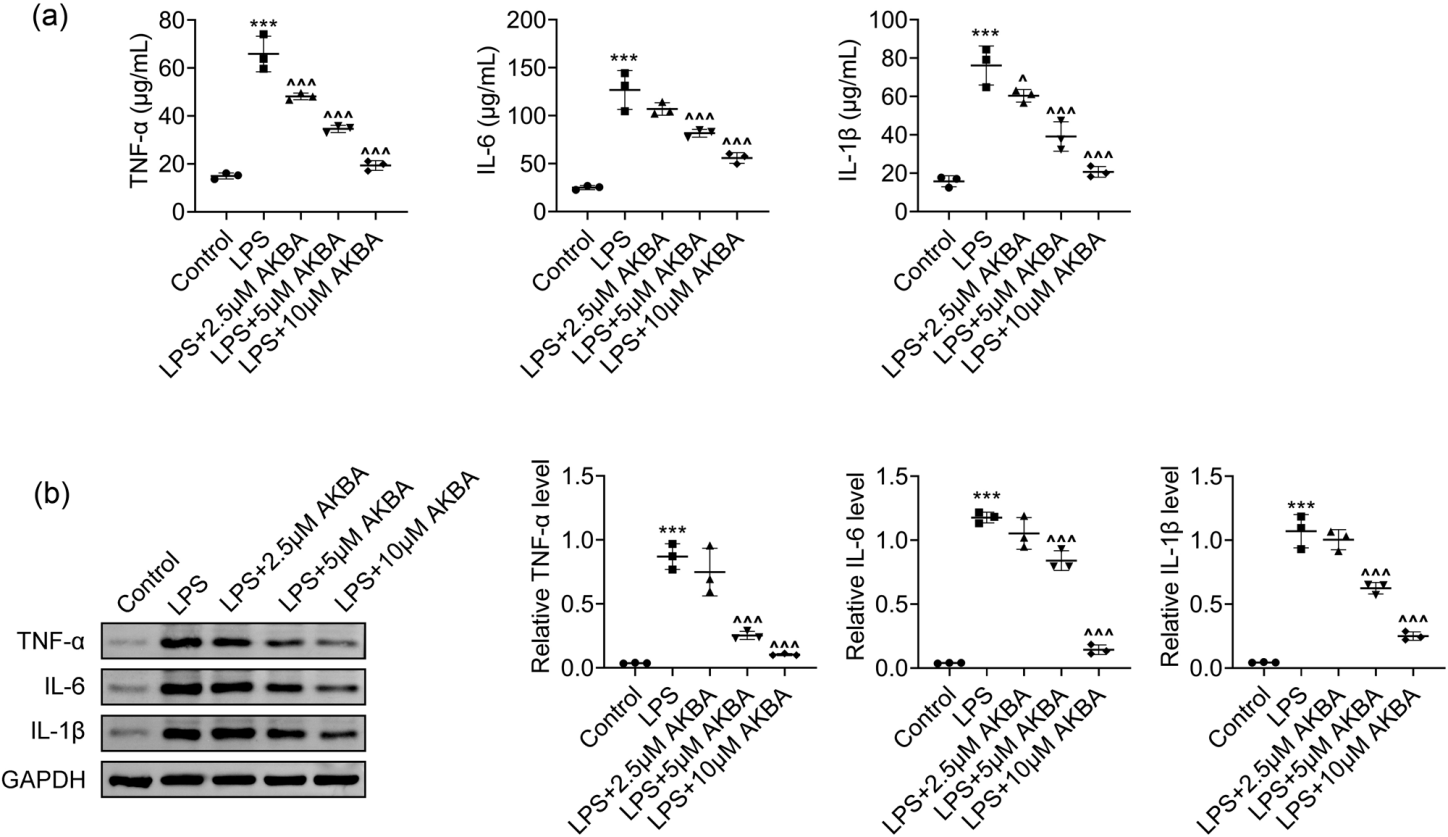
BA blocked LPS-stimulated HMEEC inflammation. (a) ELISA indicated the levels of TNF-α, IL-6, and IL-1β in HMEECs upon LPS treatment and the treatment of BA at concentrations of 2.5, 5, and 10 μM for 24 h (*n* = 3). (b) Immunoblot assays showed the expression of TNF-α, IL-6, and IL-1β in HMEECs upon LPS treatment and the treatment of BA at concentrations of 2.5, 5, and 10 μM for 24 h. The relative expression levels were quantified (*n* = 3). ***p < 0.001, LPS vs control, ^*p* < 0.05, ^^^*p* < 0.001, LPS+AKBA vs LPS. AKBA, BA.

Subsequently, the effects of BA on the inflammation of LPS-stimulated HMEECs were investigated using ELISA. It was observed that LPS treatment increased the secretion levels of inflammatory factors, suggesting the stimulation of inflammation. Conversely, BA suppressed TNF-α, IL-6, and IL-1β secretion levels in LPS-stimulated HMEECs (Figure 3a). The expression of these factors was then detected via immunoblot. Remarkably, LPS elevated the expression levels of these factors, whereas BA significantly decreased the levels of these factors, indicating the suppression of inflammation (Figure 3b). Therefore, BA suppressed the inflammation in LPS-treated HMEECs.

### 3.3 BA suppressed the NF-κB/IκBα axis in LPS-stimulated HMEECs

**Figure 4:**
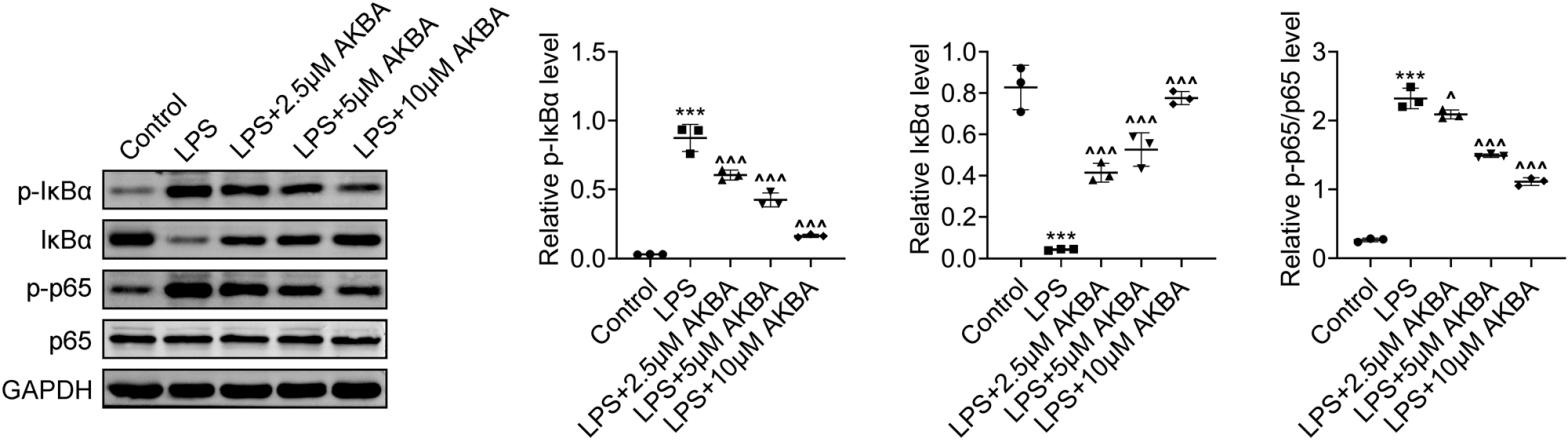
BA suppressed the NF-κB/IκBα axis in LPS-stimulated HMEECs. Immunoblot assays showed the expression and phosphorylation levels of p65 and IκBα in HMEECs upon LPS treatment and the treatment of BA at concentrations of 2.5, 5, and 10 μM for 24 h. The relative phosphorylation levels were quantified (*n* = 3). ***p < 0.001, LPS vs control, ^*p* < 0.05, ^^^*p* < 0.001, LPS+AKBA vs LPS. AKBA, BA.

The potential mechanism through which BA inhibits the progression of OM *in vitro* was investigated in the study. The impact of BA on the NF-κB/IκBα axis, pivotal in mediating cell apoptosis and inflammation, was elucidated through immunoblot analysis. Our observations revealed that LPS increased the phosphorylation levels of p65 and IκBα (Figure 4). However, subsequent treatment with BA notably reduced the phosphorylation levels of these factors in LPS-stimulated HMEECs, suggesting an inhibition of the NF-κB/IκBα pathway (Figure 4). Therefore, BA inhibited the NF-κB/IκBα axis in LPS-stimulated HMEECs.

## 4 Discussion

OM represents a prevalent inflammatory condition of the middle ear, predominantly affecting pediatric populations [17]. This condition is characterized by significant host immune responses, among other pathophysiological mechanisms [2,17]. *Streptococcus pneumoniae* is identified as the most predominant pathogen associated with OM [3,4]. The increasing prevalence of antibiotic-resistant pathogens highlights the need for alternative therapeutic strategies [6,17]. Vaccination and public health measures are paramount in preventing OM [6,17].

To enhance patient outcomes, an in-depth understanding of the underlying pathogenesis and the identification of novel therapeutic interventions are imperative. This research focused on evaluating the anti-inflammatory and anti-apoptotic efficacies of BA in HMEECs subjected to LPS, a widely accepted model for simulating conditions akin to OM. The results of our investigation elucidate that BA substantially inhibits LPS-induced inflammatory responses and apoptotic processes in HMEECs by modulating the NF-κB/IκBα signaling axis, highlighting its potential as a therapeutic agent in the management of OM.

In this study, comprehensive statistical analyses were conducted to ensure the reliability of our results. Each experiment was performed in triplicate, and results were reported as mean ± SD. The differences among groups were evaluated utilizing ANOVA, followed by *post-hoc* multiple comparison tests, setting the threshold for statistical significance at *p* < 0.05. To minimize the potential confounding factors, we meticulously ensured the consistency of cell culture conditions and treatment protocols, incorporating control groups such as untreated, LPS-only, and BA-only for comprehensive analysis. Our findings substantiate the potency of BA in reducing LPS-induced inflammation and apoptosis within HMEECs. Nevertheless, to ascertain the broader applicability of these findings, further studies, particularly *in vivo* experiments, are essential. Subsequent research will also delve into examining a broader spectrum of BA concentrations and elongated temporal intervals to elucidate its effects more comprehensively.

BA, a bioactive compound extracted from the resin of the Boswellia tree, has gained significant interest for its potential therapeutic applications across a spectrum of inflammatory conditions [18]. The mechanism underlying its anti-inflammatory efficacy is predominantly linked to the inhibition of key enzymatic pathways involved in the generation of pro-inflammatory mediators, such as leukotrienes [19]. Moreover, BA has been demonstrated to modulate the immune response through its impact on cytokine expression and the inhibition of transcription factors activation, notably NF-κB, which is pivotal in the inflammatory processes associated with OM [20,21]. Considering its favorable safety profile and efficacy in inhibiting inflammation, BA offers a promising potential as an alternative or adjunctive therapy in the management of OM. This is particularly relevant in cases where conventional treatments face limitations due to antibiotic resistance or the occurrence of adverse effects.

This study primarily focused on examining the effects of BA over a 24 h period. It is crucial to highlight that this duration is frequently employed in analogous *in vitro* studies to evaluate the initial anti-inflammatory and anti-apoptotic responses. Prior research involving LPS-stimulated HMEECs has indicated that substantial shifts in inflammation-related markers and cell viability are observable within 24 h. Nonetheless, we acknowledge that OM may persist for extended periods, especially in chronic instances. Consequently, additional research is imperative to investigate the effects of BA over longer durations, such as 48 and 72 h, to comprehensively understand its long-term therapeutic efficacy.

Our results indicated that LPS stimulation significantly increased the expression of pro-inflammatory cytokines, including TNF-α, IL-6, and IL-1β, in HMEECs. These cytokines play a pivotal role in the inflammatory cascade associated with OM by attracting immune cells and intensifying tissue damage. Conversely, the administration of BA has been shown to effectively decrease the levels of these cytokines, suggesting its potential therapeutic efficacy in inhibiting inflammatory responses in OM.

Apoptosis significantly contributes to the pathogenesis of OM by promoting excessive cell death, which compromises the structural integrity and barrier function of the middle ear epithelium [22]. Our investigations revealed that LPS exposure induces apoptosis in HMEECs, as evidenced by an increase in the pro-apoptotic protein Bax and a decrease in the anti-apoptotic protein Bcl-2. Moreover, activation of caspase-3, a critical executor of apoptosis, was observed in cells treated with LPS. Notably, treatment with BA inhibited these effects, indicating its protective role against LPS-induced apoptosis in HMEECs.

The NF-κB axis is pivotal in mediating inflammatory responses in OM through its regulation of the expression of various pro-inflammatory cytokines and adhesion molecules in response to bacterial components such as LPS [23]. The anti-inflammatory and anti-apoptotic properties of BA have been attributed to its modulation of the NF-κB/IκBα axis. Activation of the NF-κB axis by LPS stimulation is characterized by the increased phosphorylation of the p65 subunit [24]. Conversely, BA treatment inhibits the activation of NF-κB and stabilizes IκBα, thereby obstructing the transcription of pro-inflammatory and pro-apoptotic genes.

## 5 Conclusion

In conclusion, this research demonstrates that BA possesses anti-inflammatory and anti-apoptotic properties in LPS-stimulated HMEECs through the modulation of the NF-κB/IκBα pathway. These results indicate that BA could serve as a promising therapeutic agent for OM treatment. Given the well-documented anti-inflammatory capabilities of BA, it emerges as a viable candidate for OM management, particularly through its influence on key inflammatory pathways such as NF-κB. To confirm the therapeutic potential of BA for OM, further *in vivo* studies and clinical trials are essential to assess its efficacy, safety profile, and optimal dosage comprehensively.
